# Exploring the Association between Anxiety, Depression, and Gut Microbiota during Pregnancy: Findings from a Pregnancy Cohort Study in Shijiazhuang, Hebei Province, China

**DOI:** 10.3390/nu16101460

**Published:** 2024-05-12

**Authors:** Ruixin Chi, Muxia Li, Man Zhang, Na Zhang, Guohua Zhang, Lijun Cui, Guansheng Ma

**Affiliations:** 1 Department of Nutrition and Food Hygiene, School of Public Health, Peking University, 38 Xue Yuan Road, Haidian District, Beijing 100191, China; crx@bjmu.edu.cn (R.C.); zhangna@bjmu.edu.cn (N.Z.); 2 Department of Scientific Research, Beijing Children’s Hospital, Capital Medical University, National Center for Children’s Health, Beijing 100045, China; lmuxia91@126.com; 3 School of Nursing, Peking University, 38 Xue Yuan Road, Haidian District, Beijing 100191, China; zhangman@bjmu.edu.cn; 4 The Third Department of Obstetrics, Shijiazhuang Obstetrics and Gynecology Hospital, Shijiazhuang 050011, China; zghh_456@163.com; 5 The Seventh Department of Obstetrics, Shijiazhuang Obstetrics and Gynecology Hospital, Shijiazhuang 050011, China; alex_jiahao_wang@hotmail.com; 6 Laboratory of Toxicological Research and Risk Assessment for Food Safety, Peking University, 38 Xue Yuan Road, Haidian District, Beijing 100191, China

**Keywords:** gut microbiota, anxiety, depression, pregnancy

## Abstract

Negative emotions and gut microbiota during pregnancy both bear significant public health implications. However, the relationship between them has not been fully elucidated. This study, utilizing data from a pregnancy cohort, employed metagenomic sequencing to elucidate the relationship between anxiety, depression, and gut microbiota’s diversity, composition, species, and functional pathways. Data from 87 subjects, spanning 225 time points across early, mid, and late pregnancy, were analyzed. The results revealed that anxiety and depression significantly corresponded to lower alpha diversity (including the Shannon entropy and the Simpson index). Anxiety and depression scores, along with categorical distinctions of anxiety/non-anxiety and depression/non-depression, were found to account for 0.723%, 0.731%, 0.651%, and 0.810% of the variance in gut-microbiota composition (*p* = 0.001), respectively. Increased anxiety was significantly positively associated with the abundance of *Oscillibacter* sp. *KLE 1745*, *Oscillibacter* sp. *PEA192*, *Oscillibacter* sp. *KLE 1728*, *Oscillospiraceae bacterium VE202 24*, and *Treponema socranskii*. A similar association was significantly noted for *Oscillibacter* sp. *KLE 1745* with elevated depression scores. While EC.3.5.3.1: arginase appeared to be higher in the anxious group than in the non-anxious group, vitamin B12-related enzymes appeared to be lower in the depression group than in the non-depression group. The changes were found to be not statistically significant after post-multiple comparison adjustment.

## 1. Introduction

Globally, the prevalence of perinatal anxiety is approximately 15.2% [1], while the prevalence of perinatal depression is around 11.9% [2]. The perinatal period is a crucial time for the onset of mental health disorders. Mental health disorders during pregnancy are closely linked to adverse health outcomes for both the mother and the offspring. They may lead to an increased risk of suicide, substance-use disorders, breastfeeding difficulties, and challenges in forming intimate relationships with their children [3]. In addition, studies have shown that, even without a full clinical diagnosis of a mental health disorder, prenatal emotional symptoms may be associated with behavioral and neurodevelopmental problems in offspring [4]. Consequently, any degree of anxiety and depression represents a critical frontier in women-centered healthcare [5]. However, despite current research elucidating that negative emotions during pregnancy may have a negative impact on maternal and infant health outcomes, the current biological understanding of perinatal negative emotions is still insufficient [6,7].

Imbalances in the gut microbiota are associated with the occurrence of various diseases, including chronic inflammatory bowel disease, metabolic syndrome, autoimmune diseases, allergic diseases, cardiovascular diseases, neurological disorders, and tumors [8]. In addition, changes in the gut microbiota may also affect pregnancy-related diseases [9] and pregnancy outcomes for pregnant women [10]. Therefore, understanding the characteristics and influencing factors of perinatal gut microbiota is crucial for developing appropriate and individualized strategies during the maternal period. These strategies play a crucial role in reducing the long-term risk of chronic metabolic diseases, holding significant implications for public health [11,12].

Previous research suggests the possibility of a bidirectional communication mechanism between gut microbiota and the brain [13,14]. Numerous studies have corroborated the association between gut-microbiota composition and mental well-being [15]. However, inconsistencies persist regarding the correlation between anxiety, depression, and gut microbial alpha and beta diversity [16]. Notably, taxonomic investigations into anxiety and depression with sufficient resolution to discern species-level microbial differences are scarce. A previous study demonstrated that individuals with major depressive disorder tend to have higher relative abundances of *Oscillibacter valericigenes*, *Megasphaera elsdenii*, *Clostridium saccharolyticum*, *Bifidobacterium longum*, *Bifidobacterium dentium*, *Bifidobacterium adolescentis*, and *Acidaminococcus intestini* compared to a control group [16]. Conversely, the presence of *Clostridium XIVa* and *Megamonas* may inversely correlate with anxiety levels, while an increased abundance of *Holdemania* could be linked to higher anxiety [16]. Research in pregnant populations remains scarce, with existing evidence insufficient to conclusively delineate the phenotypic associations between gut microbial species and anxiety or depression at the species level. Prior studies, often relying on 16S sequencing, have typically reported associations at the genus level and above, identifying *Intestinibacter* and *Escherichia Shigella* as potential protective factors against prenatal depression, whereas *Tyzzerella* and unclassified members of the *Ruminococcaceae* family may pose an increased risk [17]. Moreover, a decline in *Erysipelatoclostridium* abundance correlates with more severe depressive symptoms [18]. Pregnant women experiencing anxiety have been found to have greater relative abundances of *Acetitomaculum*, *Acidaminococcus*, *Oxalobacter*, *Rothia*, and *Staphylococcus* [19].

Overall, current research on the interplay between negative emotions and gut microbiota is predominantly conducted in populations at non-specific physiological stages, with a paucity of studies focusing on pregnant women. Moreover, the findings regarding this association are notably inconsistent. Additionally, due to methodological constraints, existing studies are unable to provide comprehensive data at the species level. Considering the unique physiological changes during pregnancy, the results obtained from other populations may not be directly applicable to pregnant women.

In an endeavor to bridge this significant gap in knowledge, our study utilized a cohort during pregnancy, employed metagenomic sequencing, and applied epidemiological and bioinformatics analytical methods to elucidate the relationship between anxiety, depression, and gut microbiota’s diversity, composition, species, and functional pathways. The hypothesis of this study is that there is a certain degree of correlation between the gut microbiota during pregnancy and negative emotions, including anxiety and depression. The aim of our research is to enhance the understanding of the interplay between anxiety and depression and the gut microbiome of pregnant women. The research findings may provide valuable insights into the precise management of maternal mental and physical health during pregnancy.

## 2. Materials and Methods

### 2.1. Ethics Approval

This study was approved by the Biomedical Ethics Committee of Peking University (Ethics Review Approval Number: IR0001052-19150). The study was conducted in accordance with the principles of the Declaration of Helsinki. Prior to the commencement of the study, the researchers provided a comprehensive explanation of the research objectives and experimental procedures to potential participants. Following these discussions, all subjects were required to read and fully understand the informed consent form before agreeing to participate in the study and being formally enrolled.

### 2.2. Study Design

**Figure 1:**
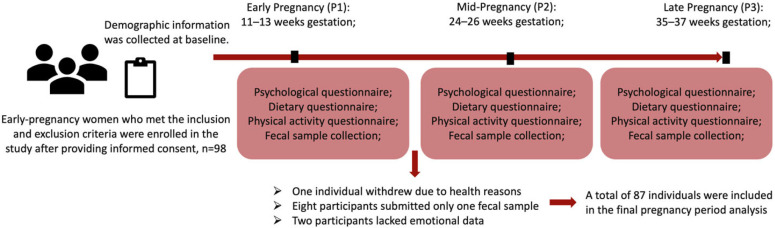
Experimental flowchart.

The study is based on a pregnancy cohort collected in Shijiazhuang, Hebei Province. The cohort study aimed to explore the factors influencing the gut microbiome by collecting intestinal microbiota data and other relevant health information from participants during early, mid, and late pregnancy. From September 2020 to April 2022, the research team recruited participants from healthy early pregnant women who were registered at the Obstetrics and Gynecology Hospital in Shijiazhuang, Hebei Province. The experimental procedure can be found in Figure 1. Ninety-eight participants, meeting the predefined inclusion and exclusion criteria, were successfully enrolled in this study. The study participants completed psychological questionnaires, dietary surveys, and physical activity questionnaires and provided fecal samples during three different pregnancy stages: P1 (weeks 11–13 of gestation during early pregnancy), P2 (weeks 24–26 of gestation during mid-pregnancy), and P3 (weeks 35–37 of gestation during late pregnancy).

### 2.3. Population

The inclusion criteria for this cohort were as follows: (1) women aged between 18 and 35 years; (2) individuals with medical records and regular prenatal check-ups at the Obstetrics and Gynecology Hospital in Shijiazhuang, Hebei Province, starting from the first trimester (11–13 weeks); (3) participants of Han ethnicity; (4) those who conceived naturally; and (5) individuals willing to be followed-up until the third trimester (35–37 weeks). The exclusion criteria encompassed: (1) individuals with infectious diseases such as AIDS, active hepatitis, syphilis, etc.; (2) those with chronic conditions, including mental illness, cardiovascular disease, kidney disease, pre-pregnancy digestive system disorders, and other ailments; (3) cases of multiple pregnancies; and (4) individuals who had used antibiotics and/or microbial preparations in the preceding month. At the analysis stage of this study, based on the main research purpose, women who had at least two fecal samples and emotional data at the same time point were included.

### 2.4. Measurements

#### 2.4.1. Basic Information

Upon enrollment, the research subjects were required to complete a questionnaire about basic information. Baseline information included age, parity, height, pre-pregnancy weight, history of smoking, and alcohol consumption before pregnancy. The pre-pregnancy BMI (body mass index) is calculated by dividing the pre-pregnancy weight (in kilograms) by the square of their height (in meters). It is categorized according to Chinese BMI standards: underweight (<18.5 kg/m^2^), normal (18.5–23.9 kg/m^2^), overweight (24.0–27.9 kg/m^2^), and obesity (≥28 kg/m^2^) [20].

#### 2.4.2. Measurement of Anxiety

Participants completed the Self-Rating Anxiety Scale (SAS) during early, mid, and late pregnancy without direct guidance from researchers. The Self-Rating Anxiety Scale (SAS) was employed to assess anxiety levels in pregnant women over the preceding month. The SAS is a globally recognized psychometric scale that comprises 20 questions, each scored on a range from 1 to 4 [21]. In China, the prevalent scoring norm involves standardizing the standardized score by multiplying the total score of the 20 items by 1.25. Participants with scores below 50 were always categorized as having no anxiety, whereas those achieving scores of 50 or higher were considered to experience anxiety [22,23,24]. And this scale has been used in the Chinese pregnant population [22]. In this study, to fully utilize the emotional data, we analyzed the anxiety scores as continuous numerical variables. Furthermore, based on whether their scores reached the anxiety threshold, the population was categorized into anxiety and non-anxiety groups, treating this as a binary variable.

#### 2.4.3. Measurement of Depression

The Self-Rating Depression Scale (SDS) was employed in the study to assess participants’ depressive symptoms. The SDS was used to assess depressive symptoms over the past month. It has been proven to be a valuable and effective tool for detecting depressive symptoms worldwide [25,26]. The SDS also includes 20 items, each scored on a 4-point scale, with the Chinese standard score equal to the total score of the 20 items multiplied by 1.25. Standard scores below 53 always indicate that depressive symptoms are within the normal range [27,28,29]. This scale has also been used in the Chinese pregnant population [27]. Similarly, in this study, we treated the depression scores as continuous numerical variables. In a similar manner, based on whether their scores reached the depression threshold, the population was categorized into depression and non-depression groups, treating this as a binary variable.

#### 2.4.4. Fecal Sample Collection

Before the commencement of the experiment, the research subjects were trained and reminded of the precautions before each follow-up. The research subjects were required to collect fecal samples on an empty stomach in the morning. Prior to collection, urine was first expelled, and disposable gloves and plastic bedpans were used for collection. A spoonful of the fecal sample, about the size of a soybean, was taken from the middle of the stool and placed in a sterile collection tube. The research subjects were required to hand over the samples to the researchers within 1 h of collection, and the researchers immediately stored them in a −80 °C freezer. The samples were then sent to a professional testing company for metagenomic sequencing. The Shenzhen Micro Health Gene Technology Co., Ltd. (Shenzhen, China) conducted the sample testing for this study.

#### 2.4.5. Shotgun Metagenomic Profiling

The methodology for fecal sample extraction has been mentioned in our previous article [30]. In brief, a specialized testing company performed metagenomic sequencing and data preprocessing on the fecal samples according to standardized procedures. Microbial DNA was extracted following the protocol included in the QIAamp DNA Stool Mini Kit (Qiagen, Germantown, MD, USA). The quality and quantity of the extracted DNA were measured using NanoDrop (Thermo Scientific, Waltham, MA, USA) and Qubit (Thermo Fisher Scientific, Singapore). The sizes of the DNA fragments were assessed through agarose gel electrophoresis. Subsequently, short-insert DNA libraries were generated using the NEBNext^®^ UltraTM DNA Library Prep Kit (NEB, Ipswich, MA, USA), and 2 × 150 bp paired-end sequencing was performed on the Illumina NovaSeq 6000 platform (Illumina, San Diego, CA, USA). Raw reads were filtered using Trimmomatic software (Version 0.33) to obtain high-quality sequencing data. Subsequently, Bowtie2 was employed to align the reads against the human genome sequence, effectively removing host contamination. The default alignment parameters were utilized. For metagenome assembly, MEGAHIT software (Version 1.1.2) was employed with default settings, filtering out contig sequences shorter than 300 base pairs. QUAST software (Version 2.3) was employed to evaluate the assembly results. To identify coding regions in the genome, MetaGeneMark software (Version 3.26) was used with default parameters. Redundancy was addressed using MMseq2 software (Version 11-e1a1c), applying a 95% similarity threshold and a 90% coverage threshold. Finally, Diamond software (Version 0.9.24) aligned the protein sequences from the non-redundant gene set with the Kyoto Encyclopedia of Genes and Genomes database. An e-value threshold <1 × 10^−5^ was set, and in cases of multiple alignment results (hits), the best alignment result was selected as the annotation for each sequence.

#### 2.4.6. Assessment of Other Covariates

In addition to the information included in the baseline characteristics, this study considered energy intake, physical activity, and probiotic and prebiotic consumption as covariates. Dietary information was assessed using a semi-quantitative food-frequency questionnaire (FFQ), which required study participants to report the frequency and average intake of food consumed over the past month [31]. Researchers then utilized the Chinese Food Composition Table to calculate individual energy intake based on food components [32]. The International Physical Activity Questionnaire (IPAQ) long form was employed to assess the physical activity levels of the study participants [33]. The questionnaire investigated the type, frequency, and duration of physical activity, allowing for a comprehensive assessment of physical activity intensity categorized into high, moderate, and low levels. Specifically, individuals were classified as having high physical activity levels if they engaged in various high-intensity activities for a total of 3 days per week, accumulating a total of 1500 MET-minutes or more weekly. Alternatively, they were categorized as high activity level if they participated in a combination of three intensity levels for 7 days, resulting in a weekly total of 3000 MET minutes or more. For those not meeting the high-level activity criteria, individuals were considered to have moderate physical activity levels if they engaged in at least 20 min of high-intensity activity for 3 days, or at least 30 min of moderate-intensity and/or walking activities for 5 days. Additionally, if their total activity across different intensities exceeded 5 days, with a weekly total of over 600 MET minutes, they fell into the moderate category. Finally, if none of the above criteria were met, individuals were classified as having low physical activity levels. Probiotic and prebiotic intake information was collected through questionnaires, with participants self-reporting their intake at different time points, recorded as ‘yes’ or ‘no’.

#### 2.4.7. Statistical Analysis

All statistical analyses were conducted using R software (R version 4.3.1). In this study, anxiety and depression during early, mid, and late pregnancy were considered as exposure factors in relation to the gut microbiota. Anxiety scores and depression scores served as continuous variables for analysis. Additionally, based on whether participants’ scores reached anxiety or depression thresholds, the population was categorized into anxiety/non-anxiety and depression/non-depression groups, treated as binary variables. Regarding microbiome analysis, several aspects were explored, including alpha diversity, beta diversity, species, pathways, and enzymes. For the alpha diversity component, linear mixed-effect models were employed to analyze the relationship between alpha diversity (including ACE, Chao 1, Shannon entropy, and Simpson index [34]) of the gut microbiota and levels of anxiety and depression at different time points in our subjects. The aforementioned analysis was conducted to validate the hypothesis concerning the association between negative emotions during pregnancy and the alpha diversity of the gut microbiota. To derive microbial patterns at the species level, after performing an arc-sin square-root transformation on relative species abundance, we used principal coordinate analysis based on Bray–Curtis dissimilarity [35]. A permutational multivariate analysis of variance (PERMANOVA) test was applied to assess the association of the overall microbial community with anxiety and depression scores. All tests were two-sided, and *p* < 0.05 were considered statistically significant.

MaAsLin2 (microbiome multivariable association with linear models) is a comprehensive R package commonly utilized for identifying multivariable correlations between clinical metadata and microbial meta-omics characteristics [36]. In this study, MaAsLin2 was used to investigate the associations between anxiety and depression scores and the relative abundance of microbial taxonomy and pathways. Both anxiety and depression scores were analyzed as continuous numerical variables, and they were also categorized as binary variables based on threshold scores from corresponding scales, which determined groupings into anxious/non-anxious or depressed/non-depressed categories. During the analysis, the microbiome data underwent a centered log-ratio (CLR) transformation. To maximize data utilization, refraining from further filtering species and pathways, all available data were included in the analysis. Additionally, following established practices in the literature, the threshold for false discovery rate (FDR) values is adjusted to 0.25 after FDR correction using the Benjamini–Hochberg method [37]. The aforementioned MaAslin2 analysis was utilized to verify the hypothesis that links anxiety and depression during pregnancy with specific species and functional pathways of the gut microbiota.

In the aforementioned analysis, the subject was treated as a random effect, while levels of anxiety or depression and time were considered as fixed effects in both unadjusted and adjusted models. The adjusted models also controlled for factors such as age [35], parity [38], pre-pregnancy BMI [38], history of smoking [35], alcohol drinking before pregnancy [35], energy intake [35], physical activity level [35], probiotics [35] and prebiotics intake [39].

## 3. Results

### 3.1. Characteristics of Participants

Following the inclusion and exclusion criteria, a total of 98 women in early pregnancy were recruited for the study. After providing informed consent, they were enrolled in the study and completed a basic demographic information form. Of the initial participants, one individual withdrew due to health reasons. Eight participants submitted only one fecal sample during pregnancy and were thus excluded from repeated measures analysis. Two participants lacked emotional data. Consequently, a total of 87 individuals were included in the final pregnancy-period analysis. The number of individuals who provided fecal samples during early, middle, and late pregnancy were 78, 77, and 70, respectively. Table 1 provides a detailed record of the basic information of the research subjects included at each time point. All subjects were from Shijiazhuang, Hebei Province, China, with a median age of 29 years old and an interquartile range of 5.5. Among them, 53 subjects were carrying their first child, while 34 subjects were carrying their second child during this study. The numbers of subjects who had a history of smoking and drinking were 2 and 17, respectively. The number of subjects who were underweight, normal weight, overweight, and obese before pregnancy were 5, 52, 17, and 13, respectively. The median self-reported energy intake calculated from dietary questionnaires is 1434 Kcal/d, with an interquartile range of 1019. In early, middle, and late pregnancy, 18, 17, and 5 individuals, respectively, exhibited high levels of physical activity. Additionally, 36, 34, and 32 individuals engaged in moderate physical activity, while 24, 26, and 32 individuals had low levels of physical activity. Furthermore, three, four, and two individuals consumed probiotics, and one, two, and one individual used prebiotics in each respective pregnancy stage.

### 3.2. Anxiety and Depression

During early, middle, and late pregnancy, the subjects were asked to report their anxiety and depression levels using a self-reported questionnaire. With regard to specific scores, as depicted in Table 2 and Figures S1 and S2, the median anxiety scores (interquartile range) during early, middle, and late pregnancy were 41.3 (11.0), 38.8 (13.8), and 40.0 (10.0), respectively. Similarly, the median depression scores (interquartile range) were 47.5 (15.6), 46.3 (15.0), and 43.8 (14.7), respectively. Notably, there were no significant differences in the scores for anxiety and depression across the three time points. The information regarding the anxiety/non-anxiety and depression/non-depression groupings can be found in Table 3. In the early, middle, and late stages of pregnancy, the number of individuals meeting the diagnostic criteria for anxiety were 16 (20.5%), 12 (15.6%), and 15 (21.4%), respectively. Similarly, the numbers of individuals meeting the diagnostic criteria for depression were 25 (32.1%), 17 (22.1%), and 16 (22.9%), respectively. Based on the chi-square test results, there were no significant differences in the proportions of anxiety/non-anxiety and depression/non-depression across different time points.

### 3.3. An Overview of Gut-Microbiota Findings

In this study, a comprehensive analysis revealed the presence of 76,320 distinct species within the sampled gut microbiota. The mean species count per sample was determined to be 2913. Dominating the profile in terms of relative abundance, the top-10 species were identified as *Faecalibacterium prausnitzii*, *Phocaeicola vulgatus*, *Eubacterium rectale*, *Bacteroides uniformis*, *Escherichia coli*, *Subdoligranulum* sp. APC924/74, *Bacteroides stercoris*, *Prevotella copri*, *Phocaeicola dorei*, and *Roseburia inulinivorans*. Upon stratification of subjects based on anxiety/depression scales, the anxiety group exhibited an average of 2792 species with non-zero relative abundance per sample, compared to 2941 species in the non-anxiety group. Statistical analysis indicated no significant difference between the two groups (t = −1.173, *p* = 0.246). Similarly, the depression group presented an average of 2789 species per sample, while the non-depression group had 2955 species, with the difference not reaching statistical significance (t = −1.595, *p* = 0.114). Further dissection of the data revealed that the ten most prevalent species in the anxiety group were *Phocaeicola vulgatus*, *Faecalibacterium prausnitzii*, *Escherichia coli*, *Bacteroides uniformis*, *Eubacterium rectale*, *Prevotella copri*, *Subdoligranulum* sp. APC924/74, *Phocaeicola dorei*, *Bacteroides fragilis*, and *Bacteroides stercoris*. The top ten most prevalent species for the non-anxiety group are *Faecalibacterium prausnitzii*, *Phocaeicola vulgatus*, *Eubacterium rectale*, *Bacteroides uniformis*, *Bacteroides stercoris*, *Subdoligranulum* sp. APC924/74, *Escherichia coli*, *Prevotella copri*, *Phocaeicola dorei*, and *Roseburia inulinivorans*. In the depression group, the ten species with the highest relative abundance included *Faecalibacterium prausnitzii*, *Phocaeicola vulgatus*, *Escherichia coli*, *Bacteroides uniformis*, *Eubacterium rectale*, *Prevotella copri*, *Bacteroides stercoris*, *Subdoligranulum* sp. APC924/74, *Phocaeicola dorei*, and *Bacteroides fragilis*. Contrastingly, the non-depression group was marked by a high prevalence of *Faecalibacterium prausnitzii*, *Phocaeicola vulgatus*, *Eubacterium rectale*, *Bacteroides uniformis*, *Subdoligranulum* sp. APC924/74, *Bacteroides stercoris*, *Escherichia coli*, *Phocaeicola dorei*, *Prevotella copri*, and *Roseburia inulinivorans*.

### 3.4. Alpha Diversity

Linear mixed-effects models were used to compare the differences in the alpha diversity of the gut microbiota at different time points among subjects with varying levels of anxiety and depression (Table 4 and Table 5). The results showed that, regardless of whether factors such as age, parity, pre-pregnancy BMI, smoking, alcohol, energy intake, physical activity level, probiotics, and prebiotics intake were adjusted, the anxiety score was significantly associated with the Shannon entropy. With each unit increase in the anxiety score, the expected Shannon entropy decreased by 0.009 (*p* = 0.044) in the unadjusted model. When controlling for covariates, with each unit increase in anxiety score, the expected Shannon entropy decreased by 0.011 (*p* = 0.027). When not controlling for covariates, the relationship between the anxiety score and the Simpson index was not significant (estimate = −0.001, *p* = 0.075). However, when controlling for covariates, a significant association was found between the anxiety score and the Simpson index. With each unit increase in the anxiety score, the expected Simpson index decreased by 0.001 (*p* = 0.049). Whether or not controlling for covariates, no association was found between the depression score and the alpha diversity indices at the species level (*p* > 0.05) in this study.

**Figure 2:**
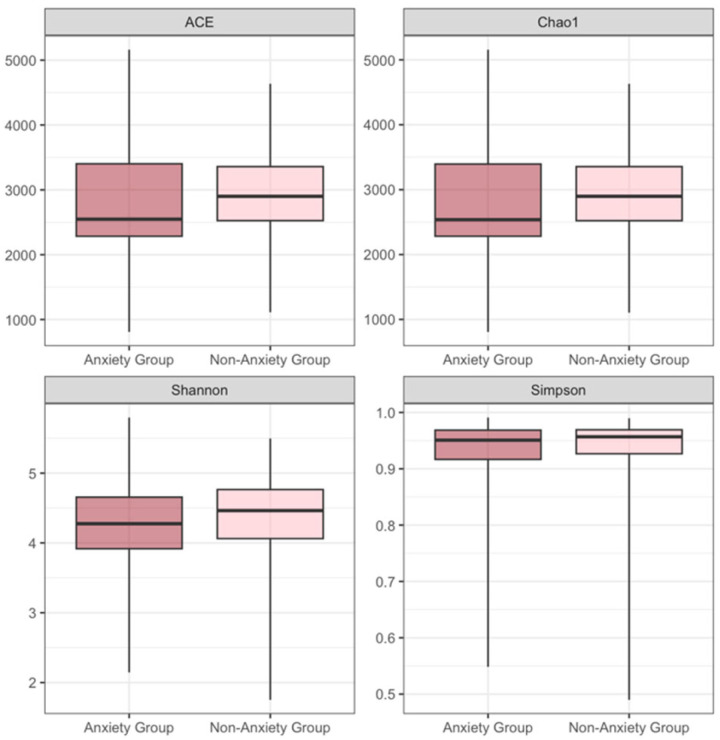
ACE, Chao 1, Shannon entropy, and Simpson index values for the anxiety and non-anxiety groups.

**Figure 3:**
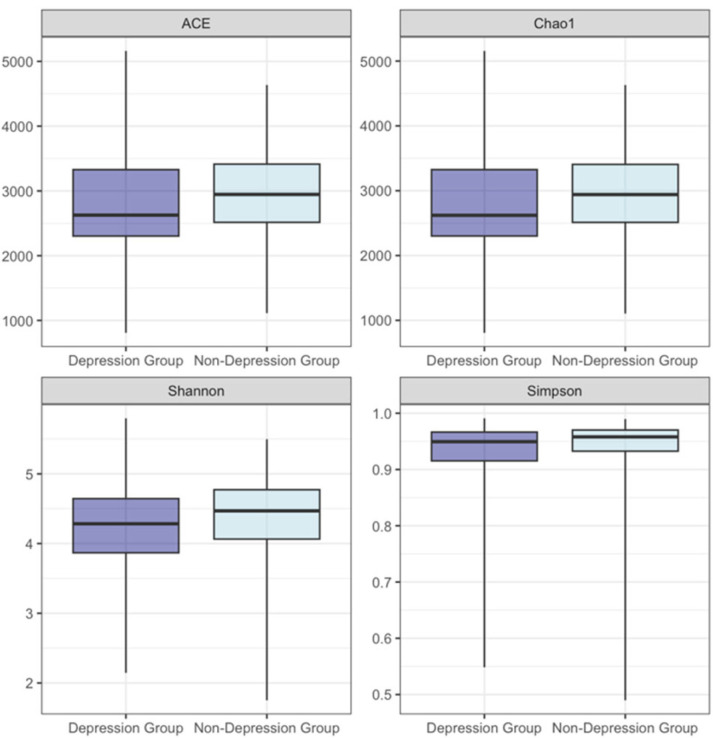
ACE, Chao 1, Shannon entropy, and Simpson index values for the depression and non-depression groups.

When anxiety scores are categorized into anxiety group/non-anxiety group and depression scores into depression group/non-depression group, the results indicate that regardless of adjusting for covariates, anxiety is significantly associated with ACE and Chao1. Compared to the non-anxiety group, the anxiety group exhibits lower levels of ACE and Chao1 (ACE: estimate = −247.762, *p* = 0.028; Chao1: estimate = −247.512, *p* = 0.028 in adjusted models). Similarly, depression is significantly associated with the Shannon entropy and the Simpson index. Compared to non-depressed individuals, the depression group has lower levels of Shannon entropy and Simpson index (Shannon entropy: estimate = −0.270, *p* = 0.014; Simpson index: estimate = −0.031, *p* = 0.027 in adjusted models). After adjusting for covariates, the depression group also exhibits lower levels in the ACE and Chao1 index compared to non-depressed individuals (ACE: estimate = −218.775, *p* = 0.036; Chao1: estimate = −218.818, *p* = 0.036). For detailed information, refer to Table 5, Figure 2 and Figure 3.

The models utilize linear mixed-effects models (LMM). In the unadjusted models, the subject is treated as a random effect, and apart from emotional scores, the model considers the impact of different time points. In the adjusted models, in addition to the factors mentioned above, possible covariates such as age, parity, pre-pregnancy BMI, history of smoking, alcohol drinking before pregnancy, energy intake, physical activity level, probiotics, and prebiotics intake are also taken into account.

### 3.5. Beta Diversity

**Figure 4:**
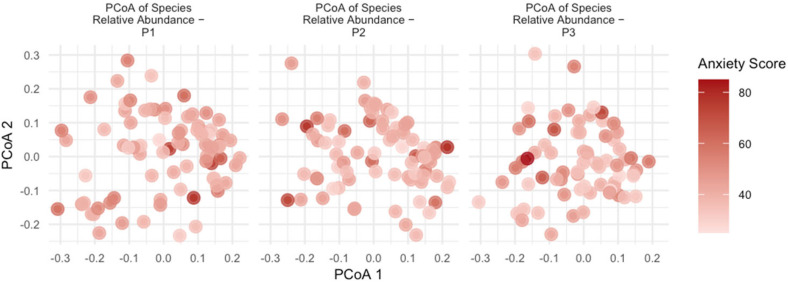
Principal coordinate analysis of the gut microbiome during early, middle, and late pregnancy, colored by anxiety score. Principal coordinate analysis of all samples based on species-level Bray–Curtis dissimilarity. Darker colors correspond to higher anxiety scores. Each point represents data from a different research subject.

**Figure 5:**
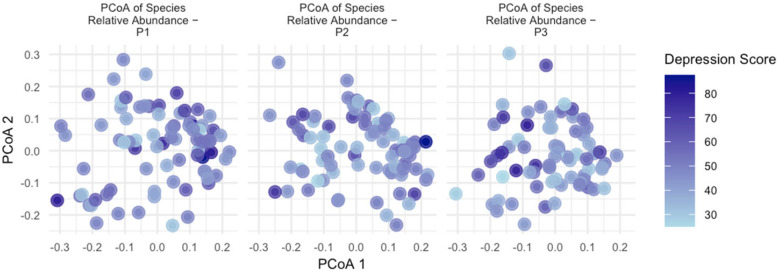
Principal coordinate analysis of the gut microbiome during early, middle, and late pregnancy, colored by depression score. Principal coordinate analysis of all samples based on species-level Bray–Curtis dissimilarity. Darker colors correspond to higher depression scores. Each point represents data from a different research subject.

Based on the species-level data, the Bray–Curtis distance was calculated, followed by a PCoA analysis. PCoA scatter plots were generated, as depicted in Figure 4 and Figure 5. These figures illustrate the distinct time points of early, middle, and late pregnancy. The depth of color represents the level of anxiety/depression, with darker colors indicating higher levels. The results of the permutational multivariate analysis of variance (PERMANOVA) model, conducted using the adonis2 function from the vegan package, showed that the anxiety score (*R*^2^ = 0.723%), depression score (*R*^2^ = 0.731%), anxiety (yes/no) (*R*^2^ = 0.651%) and depression (yes/no) (*R*^2^ = 0.810%) had a significant impact on the species composition of the gut microbiota during pregnancy (*p* = 0.001), as shown in Table 6.

### 3.6. Taxonomies and Pathways Associated with Anxiety and Depression

The analysis utilized MaAsLin2 to investigate variations in species associated with anxiety or depression. The unadjusted model considered only the impact of time point factors and subject-specific random effects, without additional covariates. Notably, at a *Q*-value threshold of less than 0.25, significant associations emerged. Higher anxiety scores correlated with an increased abundance of specific species: *Oscillibacter* sp. *KLE 1728* (estimate = 0.000013, *p* < 0.001, *Q* = 0.167), *Oscillibacter* sp. *KLE 1745* (estimate = 0.000004, *p* < 0.001, *Q* = 0.167) and *Oscillibacter* sp. *PEA192* (estimate = 0.000024, *p* < 0.001, *Q* = 0.167). Additionally, higher depression scores were associated with increased abundance of: *Butyricimonas* sp. An62 (estimate = 0.000002, *p* < 0.001, *Q* = 0.069) and *Oscillibacter* sp. *KLE 1745* (estimate = 0.000003, *p* < 0.001, *Q* = 0.131). No significant species-level differences were observed between anxious and non-anxious groups. Table S1 shows the results regarding the association between anxiety, depression, and species (including only results with *Q* < 0.25). Notably, after grouping based on anxiety or depression status, no significant species differences were observed. Consequently, this analysis only includes the results pertaining to the relationship between anxiety and depression scores and gut-microbiota species.

**Figure 6:**
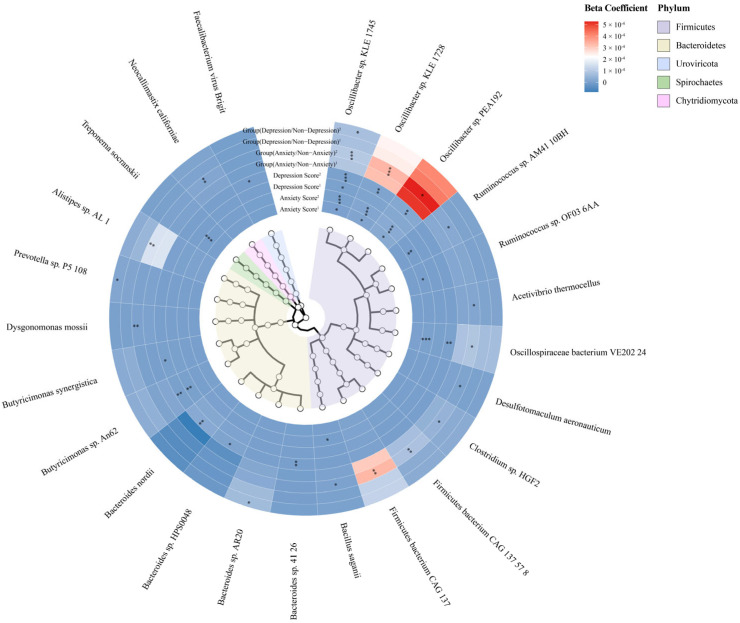
The associations between anxiety/depression and gut microbial Species. Figure 6 shows the association between filtered differentially abundant species and emotional indicators. The covariates include both unadjusted (noted as superscript1) and adjusted (noted as superscript2) models. Colors represent the strength of association. * 0.10 ≤ *q* < 0.25; ** 0.05 ≤ *q* < 0.10; *** *q* < 0.05.

In the adjusted model, covariates beyond time point factors and random effects were introduced. The covariates adjusted for were age, parity, pre-pregnancy BMI, smoking status, alcohol consumption, energy intake, physical activity level, and prebiotics and probiotics intake. Results from the adjusted model (at a *Q*-value threshold of less than 0.25) revealed significant associations for anxiety scores, depression scores, anxiety/non-anxiety groups, and depression/non-depression groups with multiple species (see Table S2 and Figure 6.). Notably, at a *Q*-value threshold of less than 0.05, higher anxiety scores were significantly associated with an increased abundance of the following species: *Oscillibacter* sp. *KLE 1745* (estimate = 0.0000043, *p* < 0.001, *Q* = 0.003), *Oscillibacter* sp. *PEA192* (estimate = 0.0000258, *p* < 0.001, *Q* = 0.010), *Oscillibacter* sp. *KLE 1728* (estimate = 0.0000145, *p* < 0.001, *Q* = 0.011), *Oscillospiraceae bacterium VE202 24* (estimate = 0.0000043, *p* < 0.001, *Q* = 0.018) and *Treponema socranskii* (estimate = 0.0000004, *p* < 0.001, *Q* = 0.040). Compared to the non-anxious group, the anxious group exhibited higher levels of *Oscillibacter* sp. *KLE 1745* (estimate = 0.0000977, *p* < 0.001, *Q* = 0.018) and *Oscillibacter* sp. *KLE 1728* (estimate = 0.0003350, *p* < 0.001, *Q* = 0.046). Furthermore, an increase in depression scores was associated with higher levels of *Oscillibacter* sp. *KLE 1745* (estimate = 0.0000032, *p* < 0.001, *Q* = 0.015).

No features of microbial functional pathways significantly related to anxiety or depression scores were found after applying MaAsLin2. Even after controlling for covariates in the model, this result remained unchanged. Tables S3 and S4 present the top-five pathways most correlated with anxiety/depression scores/grouping in the unadjusted model and the adjusted model (as determined by ascending *Q*-values and *p*-values), respectively. Notably, although some of the pathways exhibit a *p*-value less than 0.05, the *Q*-value does not attain statistical significance after multiple comparisons. For instance, these pathways include ko00430: taurine and hypotaurine metabolism, ko00760: nicotinate and nicotinamide metabolism, ko00565: ether lipid metabolism, and ko00591: linoleic acid metabolism.

**Figure 7:**
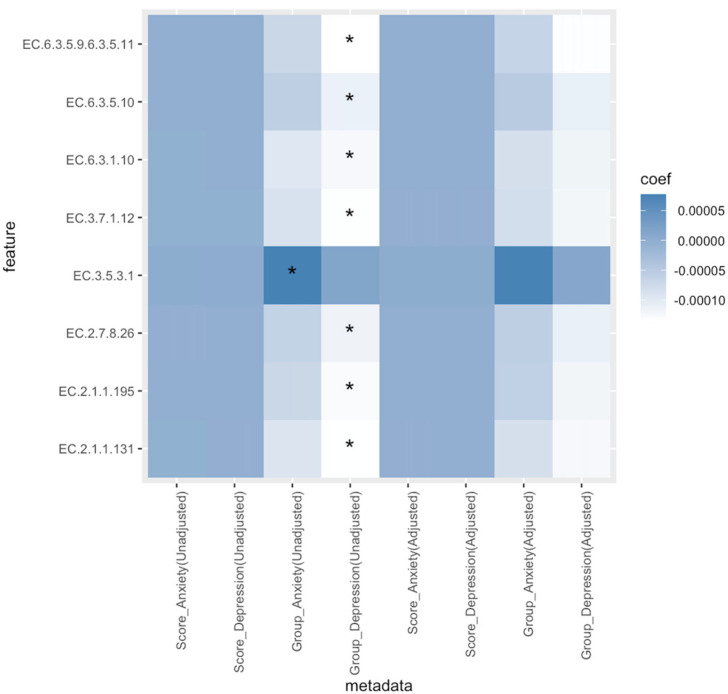
Heatmap of enzymes associated with anxiety and depression. In the unadjusted models, the subject is treated as a random effect; the model also considers the impact of different time points. In the adjusted models, in addition to the factors mentioned above, possible covariates such as age, parity, pre-pregnancy BMI, history of smoking, alcohol drinking before pregnancy, energy intake, physical activity level, probiotics, and prebiotics intake are also taken into account. * *q* < 0.25.

In terms of enzymes, when examining individual repeated measurements and time points within the model, without adjusting for other covariates, the anxious group demonstrated a greater relative abundance of EC.3.5.3.1: arginase (estimate = 0.0000772, *p* < 0.001, *Q* = 0.194) compared to the non-anxious group. Meanwhile, compared to the non-depressed group, the depressed group had lower relative abundance of EC.2.1.1.195: cobalt-precorrin-5B (C1)-methyltransferase (estimate = −0.0001280, *p* < 0.001, *Q* = 0.109), EC.2.7.8.26: adenosylcobinamide-GDP ribazoletransferase (estimate = −0.0001146, *p* < 0.001, *Q* = 0.109), EC.6.3.1.10: adenosylcobinamide-phosphate synthase (estimate = −0.0001255, *p* < 0.001, *Q* = 0.109), EC.6.3.5.9.6.3.5.11: cobyrinic acid a,c-diamide synthase (estimate = −0.0001361, *p* < 0.001, *Q* = 0.109), EC.2.1.1.131: precorrin-3B C17-methyltransferase (estimate = −0.0001353, *p* < 0.001, *Q* = 0.152), EC.6.3.5.10: adenosylcobyric acid synthase (estimate = −0.0001131, *p* < 0.001, *Q* = 0.165) and EC.3.7.1.12: cobalt-precorrin 5A hydrolase (estimate = −0.0001341, *p* < 0.001, *Q* = 0.186). The results mentioned above can be found in Figure 7 and Table S5. However, in the adjusted covariate models, the association between anxiety/depression and enzymes/pathways was not found to be statistically significant, whether based on anxiety or depression scores or in anxiety/depression groupings.

## 4. Discussion

This study finds a negative correlation between anxiety levels and certain indicators of alpha diversity in the gut microbiota of pregnant women in Shijiazhuang, Hebei Province, China. This study supports, to a certain extent, our previous hypothesis that there is a certain degree of correlation between negative emotions and gut microbiota in terms of alpha diversity, species composition, and species and functional pathways. A detailed description can be found in the following text. Our results demonstrate that anxiety scores are negatively correlated with Shannon entropy, regardless of covariate adjustment. Furthermore, anxiety scores maintain a negative correlation with the Simpson index after adjusting for covariates. Anxiety classification is significantly associated with both the ACE and Chao1 indices, with the anxious group displaying lower levels than the non-anxious group. These findings support the link between anxiety and gut microbiota diversity among pregnant women in our study population [16]. Additionally, they align with previous research suggesting that lower anxiety levels are often associated with better physical health [6,40]. Conversely, no significant association was observed between depression scores and alpha diversity of gut microbiota. This absence of association aligns with the variability seen in other populations. When using a binary classification of depression, significant differences in Shannon entropy and Simpson index emerge, with the depressed group showing lower levels than their non-depressed counterparts. This highlights the influence of diagnostic criteria and analytical methods on the interpretation of depression-related outcomes.

Furthermore, this study revealed that anxiety scores accounted for 0.723% of the variance in the species composition of gut microbiota, while depression scores accounted for 0.731% of the variance. Utilizing binary variables for anxiety/non-anxiety and depression/non-depression in our analysis, we found they explained 0.651% and 0.810% of the variance, respectively. These findings indicate that the psychological state of expectant mothers may influence the species composition of their gut microbiota, potentially impacting maternal and fetal health outcomes.

This study probed the specific gut-microbiota species correlated with anxiety and depression. Initially, without adjusting for any variables, a broad *Q*-value threshold of less than 0.25 revealed significant associations; higher anxiety scores corresponded with increased levels of *Oscillibacter* species, such as *Oscillibacter* sp. *KLE 1728*, *Oscillibacter* sp. *KLE 1745*, and *Oscillibacter* sp. *PEA192*. Concurrently, heightened depression scores were linked to an uptick in *Butyricimonas* sp. An62 and *Oscillibacter* sp. *KLE 1745*. However, these associations did not maintain significance under the more stringent *Q*-value threshold of less than 0.05. When the analysis was refined to compare the anxiety/non-anxiety and depression/non-depression groups, no significant species-level differences were detected. Yet, after adjusting for relevant covariates, a clearer picture emerged. At the stricter *Q*-value threshold, a positive correlation with anxiety scores was observed for several species, including *Oscillibacter* sp. *KLE 1745*, *Oscillibacter* sp. *PEA19*, *Oscillibacter* sp. *KLE 1728*, *Oscillospiraceae bacterium VE202 24*, and *Treponema socranskii*. Notably, the anxious group showed higher levels of *Oscillibacter* sp. *KLE 1745* and *Oscillibacter* sp. *KLE 1728* compared to their non-anxious counterparts. Additionally, an increase in depression scores was associated with elevated levels of *Oscillibacter* sp. *KLE 1745.* The association of *Oscillibacter* sp. *KLE 1745* with both anxiety and depression may suggest that different types of negative emotional states share similarities in their connections with the gut microbiota. This correlation suggests that, on one hand, the observation could offer valuable insights into the shared mechanisms that underpin the impact of diverse emotional states on microbial composition. Conversely, it may also indicate that the gut microbiota, in turn, exerts an influence on various types of negative emotions. Future research could further investigate the bidirectional relationship between emotions and gut-microbiome interactions. Moreover, the identification of such species may present potential targets for emotional management during pregnancy, opening avenues for therapeutic interventions that consider the gut–brain axis. This integrative approach could lead to more effective strategies for supporting maternal mental health and well-being.

This study addresses a significant gap by providing species-level evidence of the relationship between varying degrees of negative emotions and gut microbiota in pregnant women from Shijiazhuang, Hebei Province, China. While studies in animal models and human populations have suggested a link between the genus *Oscillibacter* and anxiety and depression, this association has not been consistently observed [41,42,43]. Although *Oscillibacter* is thought to produce short-chain fatty acids (SCFAs) like butyric acid [44], the functions of the specific species identified remain to be clarified. Our findings contribute to confirming the potential roles of these species [44]. Research has also suggested a link between Parkinson’s disease and increased levels of *Treponema socranskii* [45]. Our study adds to this by revealing a positive correlation between *Treponema socranskii* and anxiety, thus enhancing our understanding of its role in mental health. In contrast, previous research has not provided substantial psychobiological evidence connecting *Oscillospiraceae bacterium VE202 24* with mental health. A Korean study noted a reduced presence of *Oscillospiraceae* in anxious males, which was not observed in females [46]. Research from the Dutch found no significant link between negative emotions and *Oscillospiraceae* in children after adjusting for multiple tests [47]. Notably, our study contradicts these findings by identifying a positive correlation between *Oscillospiraceae bacterium VE202 24* and anxiety scores, suggesting that genus-level associations with negative affect may differ from more specific species-level associations. Variations in study populations could also contribute to these inconsistent results. The contradictory outcomes observed across different studies underscore the necessity for further research into the underlying mechanisms of these associations. Previous studies on pregnant populations have often relied on 16S sequencing methods, which do not provide detailed species-level evidence and rarely account for the broader impact of covariates, limiting comparability with our findings. A cross-sectional study involving adults aged 18–65, including 31 generalized anxiety disorder patients and 18 controls, observed higher levels of *Anaeromassilibacillus* sp. *An250* in patients with anxiety disorders [48]. However, our study did not replicate these results, highlighting the variability in findings related to gut-microbiota differences between anxious and non-anxious individuals and the importance of larger-scale, diverse cohort studies to further investigate this complex topic.

In this study, although certain pathways exhibited differences based on varying anxiety and depression scores or anxiety/depression groupings, the *p*-values were less than 0.05. However, the *Q*-values from multiple comparisons did not meet the required threshold, for instance, for pathways such as ko00430: taurine and hypotaurine metabolism, ko00760: nicotinate and nicotinamide metabolism, ko00565: ether lipid metabolism, and ko00591: linoleic acid metabolism. In the past, an animal experiment has demonstrated that, compared to their non-depressed counterparts, depressed mice exhibit significantly higher levels of ko00430: taurine and hypotaurine metabolism [49]. A past randomized controlled trial attempting to treat depression through the supplementation of probiotics and vitamin B7 revealed notable differences in the ko00760: nicotinate and nicotinamide metabolism pathway between the intervention and control groups. Although the experimental group showed an improvement in psychiatric symptoms over time, the difference was not significant in the placebo group [50]. However, the differences in these pathways were not significant after multiple comparisons in this study. But this result is also reasonable, as positive results are not always found in studies of other populations [47]. In other pathways, a previous study specifically investigating generalized anxiety disorder revealed that individuals with this condition exhibited an elevation in the aspartate degradation I module within their gut metabolism compared to the control group [48]. Additionally, a large-scale cross-sectional study conducted in the Netherlands revealed a negative correlation between major depressive disorder and the tryptophan and glutamate synthesis modules, as well as the 3,4-dihydroxyphenylacetic acid synthesis module (associated with dopamine metabolism) [51]. Furthermore, a study involving patients with inflammatory bowel diseases demonstrated that depression was associated with the central carbohydrate metabolism, pectin, and glycosaminoglycan pathways [52]. It is important to note that these studies differed from our research in terms of anxiety and depression diagnostic criteria and study populations, which may partially explain the divergent results. The study provides results from a Chinese pregnant population, which could serve as a reference for subsequent investigations. In light of these findings, the current analyses of the association between anxiety, depression, and microbial functional pathways show variations across different populations, necessitating further research to explore the reasons behind these differences. Future research can further explore different populations and further explore the possible mechanisms of the association between gut microbiota and emotions.

When stratifying the population based on the presence or absence of anxiety and depression, considering only individual repeated measurements and temporal factors without controlling for other covariates, the group with anxiety demonstrated a greater relative abundance of EC.3.5.3.1: arginase compared to normal individuals. An animal study conducted in mice demonstrated that the reduction of arginase 1-positive microglia could potentially lead to anxiety/depression-like behavior through the decrease of pCREB/BDNF in Alzheimer’s Disease [53]. A population study revealed an increase in serum arginase activity in patients with depression [54]. However, in our research, we only found a higher concentration of arginase in the gut microbiota of the anxiety group compared to non-anxious individuals, with no such relationship observed in the depression group. When controlling for other covariates, the association between arginase and the anxiety group is no longer significant.

Our research also found that, without controlling for other covariates, compared to non-depressed individuals, the depression group showed a lower relative abundance of EC.2.1.1.195: cobalt-precorrin-5B (C1)-methyltransferase, EC.2.7.8.26: adenosylcobinamide-GDP ribazoletransferase, EC.6.3.1.10: adenosylcobinamide-phosphate synthase, EC.6.3.5.9.6.3.5.11: cobyrinic acid a,c-diamide synthase, EC.2.1.1.131: precorrin-3B C17-methyltransferase, EC.6.3.5.10: adenosylcobyric acid synthase (glutamine hydrolyzing), and EC.3.7.1.12: cobalt-precorrin 5A hydrolase. This may suggest potential physiological mechanisms underlying pregnancy depression. One study showed that in children with ADHD taking psychostimulant medication, the bacterial gene abundance of EC.6.3.1.10: adenosylcobinamide-phosphate synthase was significantly reduced compared to those not using psychostimulant medication [55]. Although we did not find direct evidence of the other enzymes discovered in this study being related to depression, interestingly, EC.6.3.5.9.6.3.5.11: cobyrinic acid a,c-diamide synthase [56], EC.2.7.8.26: Adenosylcobinamide-GDP ribazoletransferase [57], EC.2.1.1.195: cobalt-precorrin-5B (C1)-methyltransferase [58], EC.2.1.1.131: precorrin-3B C17-methyltransferase [59], EC.6.3.5.10: adenosylcobyric acid synthase (glutamine-hydrolyzing) [60], and EC.3.7.1.12: cobalt-precorrin 5A hydrolase [61] are all related to the biosynthesis of vitamin B12. Previous research suggests that vitamin B12 can regulate the composition of the gut microbiota, such as enhancing SCFA-producing bacteria [62] and increasing α diversity [63]. It has also been found that lower levels of vitamin B12 in the body at the population level are associated with a higher risk of depression, and it has been suggested that supplementing vitamin B12 may be beneficial for improving depressive states [64]. The enzymes related to depression found in this study are closely related to the biosynthetic pathway of vitamin B12. Future research may further analyze this feature. It is noteworthy that when emotions were compared as scores, no significant differences were observed in the functional pathways of gut-microbiota species. However, when emotions were grouped based on whether they met the criteria for depression and anxiety, differences in functional pathways were observed. This suggests that differences in some functional pathways may only manifest at specific levels. However, it is important to note that after controlling for other relevant covariates, the relationship between enzymes and anxiety or depression is no longer significant. This suggests that enzymes related to arginase and the biosynthetic pathway of vitamin B12 may impact psychosocial factors, necessitating further in-depth exploration through subsequent research. However, these relationships do not always exist. Additionally, it is crucial to carefully consider potential confounding factors during analysis to draw relevant conclusions.

Some studies have attempted to explain the potential mechanisms underlying the relationship between gut microbiota and emotions. For instance, the brain–gut–microbiota axis may supports the bidirectional communication pathways involving the neuroendocrine and inflammatory mechanisms that exist between the gut and the brain [65,66,67]. Some studies have also pointed out that negative emotions are associated with elevated levels of cortisol and inflammatory mediators, leading to a persistent pro-inflammatory state [68,69,70]. The gut microbiota is a key regulator within the gut–brain axis, modulating the production of neurotransmitters and their precursors through bacterial species, and can secrete and upregulate the necessary proteins and metabolites involved in the release of neuropeptides and gut hormones, affecting brain function through neural, endocrine, and immune pathways [71]. Future research could refer to some of the findings in this study, such as the new discoveries at the species level and the enzymes and pathways related to the biosynthesis of vitamin B12 and arginase, to further explore the potential mechanisms of the relationship between negative emotions during pregnancy and the gut microbiota.

This study integrates approaches from psychology, microbiology, and epidemiology to investigate public health issues. Current research on the interrelation between negative emotions and gut microbiota is primarily cross-sectional [72]. However, for the pregnant population, it is imperative to consider the longitudinal context of an individual, taking into account the various stages of pregnancy. This research utilizes cohort study data, taking into account different time points and repeated measurements, to more precisely examine the relationship between negative emotions and the gut microbiota during pregnancy. In addition, employing metagenomic detection methods allows for the provision of relatively more comprehensive microbial data. What is more, we have meticulously accounted for potential confounding factors that may characterize our research cohort, including age, dietary patterns, physical activity levels, smoking and alcohol consumption prior to pregnancy, baseline body mass, and the consumption of probiotics and prebiotics, as well as parity. This, to a certain extent, enhances the accuracy of our model results. Moreover, it is worth noting that, because research has pointed out that even if it does not meet the diagnostic criteria, different levels of negative emotions can also have an impact on health [73]. Therefore, this study focuses on different levels of anxiety and depression, rather than simply dividing the population into anxious/non-anxious or depressed/non-depressed. This study also hopes to call on more people to pay attention to the mental health of pregnant women, whether they are diagnosed with a mental illness or not. This study focuses on the physical and mental health of pregnant women, not only focusing on the disease population but also on the emotional state of the normal population, which is conducive to the promotion of research results. We hope that this study will serve as a valuable reference for subsequent research to further understand the physiological mechanisms of negative emotions during pregnancy. This could further contribute to the management of negative emotions during pregnancy, enhance the childbirth experiences of pregnant women, and improve their physical and mental health.

This study has certain limitations in the following aspects. Participants in our study are all from Shijiazhuang, Hebei Province, China, which limits the inference of the results to a wider population [74,75,76]. Additionally, in this study, anxiety and depression were measured using the Self-Rating Anxiety Scale and the Self-Rating Depression Scale, respectively. Although these scales are internationally recognized and commonly utilized, they have been repeatedly employed within the Chinese pregnant population and are widely applied in epidemiological surveys due to their ease of completion [22,27,77,78]. However, these scales do not provide a detailed subdivision of anxiety and depression, such as social phobia, panic disorder, or dysthymia [51]. Moreover, in terms of emotional data measurement, the scales we used were not specifically designed for pregnant populations. Although they have been used among pregnant women in China, this may impose certain limitations on the assessment of negative emotions in this population [77,78]. In addition, this study only conducts association analysis on negative emotions and gut microbiota during pregnancy. In the future, it should be combined with more blood biochemical indicators, etc., to further explore the possible mechanisms of association. Multi-center research is also expected, which can have a larger sample to further explore the relationship between negative emotions during pregnancy and gut microbiota, and further examine the universality of these findings for populations with different biological or demographic characteristics. Furthermore, a study has indicated that the composition, rather than the diversity, of the oral microbiome is associated with symptoms of anxiety and depression in adolescents [79]. Our research on the gut microbiome of the pregnant population suggests that both the composition and diversity of the microbiome are related to symptoms of anxiety and depression. This implies that future research could delve deeper into different populations and various microbiome sources.

A deeper comprehension of the relationship between gut microbiota and anxiety and depression could facilitate the development of targeted intervention strategies to enhance psychophysical health during pregnancy [80,81]. Future research may build upon the findings of this study to further investigate this domain. It is hoped that more studies will focus on the psychophysical health issues of the prenatal period.

## 5. Conclusions

In pregnant women from Shijiazhuang, China, anxiety and depression are associated with gut-microbiota diversity, overall species composition, and specific species. Compared to non-anxious individuals, the anxiety group exhibits higher levels of the enzyme arginase, while the depression group shows lower levels of an enzyme related to vitamin B12 synthesis than the non-depression group. However, after adjusting for covariates, these associations are no longer significant. Future research endeavors could further elucidate the underlying mechanisms, thereby facilitating the development of more effective strategies for managing maternal mental and physical well-being.

## Figures and Tables

**Table 1:** Basic information of participants.

Variable	Level	P1		P2		P3		Overall	
		N	Percentage	N	Percentage	N	Percentage	N	Percentage
Age *	Median (IQR)	29 (5.75)		29 (6)		29 (6)		29 (5.5)	
Pre-Pregnancy BMI	Underweight	5	6.4%	5	6.5%	4	5.7%	5	5.0%
	Normal	47	60.3%	45	58.4%	10	14.3%	52	52.0%
	Overweight	15	19.2%	16	20.8%	42	60.0%	17	17.0%
	Obesity	11	14.1%	11	14.3%	14	20.0%	13	13.0%
Parity	1	47	60.3%	50	64.9%	44	62.9%	53	53.0%
	2	31	39.7%	27	35.1%	26	37.1%	34	34.0%
Smoking	Yes	2	2.6%	2	2.6%	1	1.4%	2	2.0%
	No	76	97.4%	75	97.4%	69	98.6%	85	85.0%
Alcohol	Yes	15	19.2%	15	19.5%	13	18.6%	17	17.0%
	No	63	80.8%	62	80.5%	57	81.4%	70	70.0%
Energy Intake *	Median (IQR)	1279 (992)		1417 (961)		1730 (1257)		1434 (1019)	
Physical Activity Level	High	18	23.1%	17	22.1%	5	7.1%	40	17.8%
	Median	36	46.2%	34	44.2%	32	47.1%	103	45.8%
	Low	24	30.8%	26	33.8%	32	45.7%	82	36.4%
Probiotics	Yes	3	3.8%	4	5.2%	2	2.9%	9	4.0%
	No	75	96.2%	73	94.8%	68	97.1%	216	96.0%
Prebiotics	Yes	1	1.3%	2	2.6%	1	1.4%	4	1.8%
	No	77	98.7%	75	97.4%	69	98.6%	221	98.2%
Total		78		77		70		87	

* Age and energy intake are represented by the median and interquartile range (IQR). The unit of age is years, and the unit of energy intake is kcal/d. Other indicators are presented as counts (percentages). Age, pre-pregnancy BMI, parity, smoking, and alcohol describe the status of study participants at the baseline survey. Energy intake, physical activity level, probiotics, and prebiotics describe the actual conditions of the study participants at that time point.

**Table 2:** Anxiety and depression scores of the study subjects at different time points.

	P1	P2	P3	χ^2^ *	*p*
Anxiety Score	41.3 (11.0)	38.8 (13.8)	40.0 (10.0)	4.607	0.100
Depression Score	47.5 (15.6)	46.3 (15.0)	43.8 (14.7)	3.704	0.157

* The anxiety and depression scores of the study participants were assessed at three time points, represented by the median (interquartile range). The Kruskal–Wallis rank sum test was used to assess anxiety and depression scores at three different time points.

**Table 3:** Distribution of anxiety and depression groups at different time points.

Group	P1		P2		P3		Overall		χ^2^ *	*p*
	N	Percentage	N	Percentage	N	Percentage	N	Percentage		
Anxiety Group	16	20.5	12	15.6	15	21.4	43	19.1	0.960	0.600
Non-Anxiety Group	62	79.5	65	84.4	55	78.6	182	80.9		
Depression Group	25	32.1	17	22.1	16	22.9	58	25.8	2.500	0.300
Non-Depression Group	53	67.9	60	77.9	54	77.1	167	74.2		

* The chi-square test was used to assess whether there were significant differences in anxiety/depression proportions among these three time points.

**Table 4:** Associations between anxiety and depression scores and gut-microbiota alpha diversity in research subjects: results from linear mixed-effects models.

Variable	Model	Metrics for Alpha Diversity	Estimate	Std. Error	df	t	*p*
Anxiety Score	Unadjusted	ACE	−5.454	4.516	219.260	−1.208	0.229
Anxiety Score	Adjusted	ACE	−7.137	4.660	207.118	−1.532	0.127
Anxiety Score	Unadjusted	Chao1	−5.460	4.514	219.187	−1.210	0.228
Anxiety Score	Adjusted	Chao1	−7.149	4.657	206.977	−1.535	0.126
Anxiety Score	Unadjusted	Shannon Entropy	−0.009	0.005	218.078	−2.025	0.044
Anxiety Score	Adjusted	Shannon Entropy	−0.011	0.005	207.035	−2.234	0.027
Anxiety Score	Unadjusted	Simpson Index	−0.001	0.001	215.991	−1.787	0.075
Anxiety Score	Adjusted	Simpson Index	−0.001	0.001	205.413	−1.984	0.049
Depression Score	Unadjusted	ACE	1.149	3.737	220.385	0.308	0.759
Depression Score	Adjusted	ACE	−0.838	3.833	210.214	−0.219	0.827
Depression Score	Unadjusted	Chao1	1.137	3.735	220.343	0.304	0.761
Depression Score	Adjusted	Chao1	−0.855	3.830	210.132	−0.223	0.824
Depression Score	Unadjusted	Shannon Entropy	−0.002	0.004	219.456	−0.519	0.604
Depression Score	Adjusted	Shannon Entropy	−0.004	0.004	209.868	−0.910	0.364
Depression Score	Unadjusted	Simpson Index	<0.001	<0.001	217.873	−0.884	0.377
Depression Score	Adjusted	Simpson Index	−0.001	0.001	208.589	−1.097	0.274

The models utilize linear mixed-effects models (LMM). In the unadjusted models, the subject is treated as a random effect, and apart from emotional scores, the model considers the impact of different time points. In the adjusted models, in addition to the factors mentioned above, possible covariates such as age, parity, pre-pregnancy BMI, history of smoking, alcohol drinking before pregnancy, energy intake, physical activity level, probiotics, and prebiotics intake are also taken into account.

**Table 5:** Associations between anxiety and depression grouping and gut-microbiota alpha diversity in research subjects: results from linear mixed-effects models.

Variable	Model	Metrics for Alpha Diversity	Estimate	Std. Error	df	t	*p*
Group_Anxiety	Unadjusted	ACE	−219.618	110.415	219.382	−1.989	0.048
Group_Anxiety	Adjusted	ACE	−247.762	112.065	211.760	−2.211	0.028
Group_Anxiety	Unadjusted	Chao1	−219.358	110.368	219.445	−1.988	0.048
Group_Anxiety	Adjusted	Chao1	−247.512	112.004	211.789	−2.210	0.028
Group_Anxiety	Unadjusted	Shannon Entropy	−0.149	0.116	220.490	−1.288	0.199
Group_Anxiety	Adjusted	Shannon Entropy	−0.168	0.119	211.931	−1.407	0.161
Group_Anxiety	Unadjusted	Simpson Index	−0.015	0.015	220.991	−1.032	0.303
Group_Anxiety	Adjusted	Simpson Index	−0.017	0.015	211.964	−1.147	0.253
Group_Depression	Unadjusted	ACE	−189.147	102.327	220.990	−1.848	0.066
Group_Depression	Adjusted	ACE	−218.775	103.806	211.260	−2.108	0.036
Group_Depression	Unadjusted	Chao1	−189.116	102.274	220.985	−1.849	0.066
Group_Depression	Adjusted	Chao1	−218.818	103.737	211.205	−2.109	0.036
Group_Depression	Unadjusted	Shannon Entropy	−0.233	0.106	220.889	−2.193	0.029
Group_Depression	Adjusted	Shannon Entropy	−0.270	0.110	211.444	−2.467	0.014
Group_Depression	Unadjusted	Simpson Index	−0.027	0.013	220.300	−2.049	0.042
Group_Depression	Adjusted	Simpson Index	−0.031	0.014	210.718	−2.229	0.027

Group_Anxiety refers to the binary classification results obtained by grouping individuals at a specific time point based on whether their anxiety scores meet the anxiety criteria. Group_Depression refers to the binary classification results obtained by grouping individuals at a specific time point based on whether their depression scores meet the depression criteria.

**Table 6:** Results of PERMANOVA analysis: assessing microbiota differences in subjects with varying levels of anxiety and depression.

Variable	*R* ^2^	F	*p*
Anxiety Score	0.723%	3.115	0.001
Depression Score	0.731%	3.150	0.001
Group_Anxiety	0.651%	2.808	0.001
Group_Depression	0.810%	3.497	0.001

## Data Availability

For questions regarding the acquisition of original data, collaboration, and other related matters, please contact the corresponding author via email for further communication due to privacy restrictions.

## Supplementary Materials

The following supporting information can be downloaded at https://www.mdpi.com/article/10.3390/nu16101460/s1: Figure S1: Temporal Trends in Anxiety Scores Across Multiple Time Points; Figure S2: Temporal Trends in Depression Scores Across Multiple Time Points.; Table S1: The List of Species Significantly Associated with Anxiety and Depression in an Unadjusted Model; Table S2: The List of Species Significantly Associated with Anxiety and Depression in an Adjusted Model; Table S3: Functional Pathways and Their Correlation with Anxiety/Depression Scores and Anxiety/Non-anxiety and Depression/Non-depression Groupings in an Unadjusted Model; Table S4: Functional Pathways and Their Correlation with Anxiety/Depression Scores and Anxiety/Non-anxiety and Depression/Non-depression Groupings in an Adjusted Model; Table S5: The List of Enzymes Significantly Associated with Anxiety and Depression in an Unadjusted Model.

## References

[1] Dennis C.L., Falah-Hassani K., Shiri R. (2017). Prevalence of antenatal and postnatal anxiety: Systematic review and meta-analysis. Br. J. Psychiatry.

[2] Woody C.A., Ferrari A.J., Siskind D.J., Whiteford H.A., Harris M.G. (2017). A systematic review and meta-regression of the prevalence and incidence of perinatal depression. J. Affect. Disord..

[3] Stevenson K., Fellmeth G., Edwards S., Calvert C., Bennett P., Campbell O.M.R., Fuhr D.C. (2023). The global burden of perinatal common mental health disorders and substance use among migrant women: A systematic review and meta-analysis. Lancet Public Health.

[4] Gustafsson H.C., Sullivan E.L., Nousen E.K., Sullivan C.A., Huang E., Rincon M., Nigg J.T., Loftis J.M. (2018). Maternal prenatal depression predicts infant negative affect via maternal inflammatory cytokine levels. Brain Behav. Immun..

[5] Zhang B., Qin S. (2023). Mental health at the forefront of women-centred health-care systems. Lancet.

[6] Sherer M.L., Voegtline K.M., Park H.S., Miller K.N., Shuffrey L.C., Klein S.L., Osborne L.M. (2022). The immune phenotype of perinatal anxiety. Brain Behav. Immun..

[7] Brann E., Skalkidou A., Schwarz J., Papadopoulos F.C., Sundstrom Poromaa I., Fransson E. (2022). Longitudinal assessment of inflammatory markers in the peripartum period by depressive symptom trajectory groups. Brain Behav. Immun. Health.

[8] Wu Y., Wan J., Choe U., Pham Q., Schoene N.W., He Q., Li B., Yu L., Wang T.T.Y. (2019). Interactions between Food and Gut Microbiota: Impact on Human Health. Annu. Rev. Food Sci. Technol..

[9] Lu X.W., Shi Z., Jiang L.L., Zhang S.Y. (2024). Maternal gut microbiota in the health of mothers and offspring: From the perspective of immunology. Front. Immunol..

[10] Fox C., Eichelberger K. (2015). Maternal microbiome and pregnancy outcomes. Fertil. Steril..

[11] Gensollen T., Iyer S.S., Kasper D.L., Blumberg R.S. (2016). How colonization by microbiota in early life shapes the immune system. Science.

[12] Robertson R.C., Manges A.R., Finlay B.B., Prendergast A.J. (2019). The Human Microbiome and Child Growth—First 1000 Days and Beyond. Trends Microbiol..

[13] Fung T.C. (2020). The microbiota-immune axis as a central mediator of gut-brain communication. Neurobiol. Dis..

[14] Galley J.D., Mashburn-Warren L., Blalock L.C., Lauber C.L., Carroll J.E., Ross K.M., Hobel C., Coussons-Read M., Dunkel Schetter C., Gur T.L. (2023). Maternal anxiety, depression and stress affects offspring gut microbiome diversity and bifidobacterial abundances. Brain Behav. Immun..

[15] Christian L.M. (2019). The gut microbiome and mental health: Taking baby steps. Brain Behav. Immun..

[16] Simpson C.A., Diaz-Arteche C., Eliby D., Schwartz O.S., Simmons J.G., Cowan C.S.M. (2021). The gut microbiota in anxiety and depression—A systematic review. Clin. Psychol. Rev..

[17] Fang Q., Tu Y., Fan X., Zang T., Wu N., Qiu T., Li Y., Bai J., Liu Y. (2023). Inflammatory cytokines and prenatal depression: Is there a mediating role of maternal gut microbiota?. J. Psychiatr. Res..

[18] Rajasekera T.A., Galley J.D., Mackos A.R., Chen H.J., Mitchell J.G., Kleinman J.J., Cappelucci P., Mashburn-Warren L., Lauber C.L., Bailey M.T. (2024). Stress and depression-associated shifts in gut microbiota: A pilot study of human pregnancy. Brain Behav. Immun. Health.

[19] Hechler C., Borewicz K., Beijers R., Saccenti E., Riksen-Walraven M., Smidt H., de Weerth C. (2019). Association between Psychosocial Stress and Fecal Microbiota in Pregnant Women. Sci. Rep..

[20] Zhou B.F., Coorperative Meta-Analysis Group of China Obesity Task Force (2002). Predictive values of body mass index and waist circumference for risk factors of certain related diseases in Chinese adults—Study on optimal cut-off points of body mass index and waist circumference in Chinese adults. Biomed. Environ. Sci..

[21] Zung W.W. (1971). A rating instrument for anxiety disorders. Psychosom. J. Consult. Liaison Psychiatry.

[22] Yue C.Y., Liu C.P., Wang J., Zhang M., Wu H.J., Li C.R., Yang X.L. (2021). Association between social support and anxiety among pregnant women in the third trimester during the coronavirus disease 2019 (COVID-19) epidemic in Qingdao, China: The mediating effect of risk perception. Int. J. Soc. Psychiatry.

[23] Liao M.L., Fang F., Liu G.X., Zhang Y.X., Deng C.Q., Zhang X.Q. (2020). Influencing factors and correlation of anxiety, psychological stress sources, and psychological capital among women pregnant with a second child in Guangdong and Shandong Province. J. Affect. Disord..

[24] Yue J.M., Zang X.Y., Le Y.Y., An Y.Y. (2022). Anxiety, depression and PTSD among children and their parent during 2019 novel coronavirus disease (COVID-19) outbreak in China. Curr. Psychol..

[25] Zung W.W. (1965). A self-rating depression scale. Arch. Gen. Psychiatry.

[26] Biggs J.T., Wylie L.T., Ziegler V.E. (1978). Validity of the Zung self-rating depression scale. Br. J. Psychiatry.

[27] Zhou Y., Huang J.G., Baker P.N., Liao B.Z., Yu X.Y. (2022). The prevalence and associated factors of prenatal depression and anxiety in twin pregnancy: A cross-sectional study in Chongqing, China. BMC Pregnancy Childbirth.

[28] Chen Q.Q., Lin F.M., Chen D.H., Ye Y.M., Gong G.M., Chen F.F., Huang S.F., Peng S.L. (2023). Analysis of mental health status and related factors in patients with acute cerebral infarction. World J. Psychiatry.

[29] Wang Y.N., Di Y., Ye J.J., Wei W.B. (2021). Study on the public psychological states and its related factors during the outbreak of coronavirus disease 2019 (COVID-19) in some regions of China. Psychol. Health Med..

[30] Li M.X., Zhang G.H., Cui L.J., Zhang L., Zhou Q., Mu C.X., Chi R.X., Zhang N., Ma G.S. (2023). Dynamic changes in gut microbiota during pregnancy among Chinese women and influencing factors: A prospective cohort study. Front. Microbiol..

[31] Shahar D., Shai I., Vardi H., Brener-Azrad A., Fraser D. (2003). Development of a semi-quantitative Food Frequency Questionnaire (FFQ) to assess dietary intake of multiethnic populations. Eur. J. Epidemiol..

[32] Prevention CCFDCA (2009). Chinese Food Composition Table.

[33] Fan M., Lyu J., He P. (2014). Chinese guidelines for data processing and analysis concerning the International Physical Activity Questionnaire. Zhonghua Liu Xing Bing Xue Za Zhi.

[34] Feranchuk S., Belkova N., Potapova U., Kuzmin D., Belikov S. (2018). Evaluating the use of diversity indices to distinguish between microbial communities with different traits. Res. Microbiol..

[35] Li Y.P., Wang D.D., Satija A., Ivey K.L., Li J., Wilkinson J.E., Li R.F., Baden M., Chan A.T., Huttenhower C. (2021). Plant-Based Diet Index and Metabolic Risk in Men: Exploring the Role of the Gut Microbiome. J. Nutr..

[36] Mallick H., Rahnavard A., McIver L.J., Ma S., Zhang Y., Nguyen L.H., Tickle T.L., Weingart G., Ren B., Schwager E.H. (2021). Multivariable association discovery in population-scale meta-omics studies. PLoS Comput. Biol..

[37] Wang K., Mehta R.S., Ma W., Nguyen L.H., Wang D.D., Ghazi A.R., Yan Y., Al-Shaar L., Wang Y., Hang D. (2023). The gut microbiome modifies the associations of short- and long-term physical activity with body weight changes. Microbiome.

[38] Kennedy K.M., Plagemann A., Sommer J., Hofmann M., Henrich W., Barrett J.F.R., Surette M.G., Atkinson S., Braun T., Sloboda D.M. (2023). Parity modulates impact of BMI and gestational weight gain on gut microbiota in human pregnancy. Gut Microbes.

[39] Lordan C., Thapa D., Ross R.P., Cotter P.D. (2020). Potential for enriching next-generation health-promoting gut bacteria through prebiotics and other dietary components. Gut Microbes.

[40] Mareckova K., Marecek R., Jani M., Zackova L., Andryskova L., Brazdil M., Nikolova Y.S. (2023). Association of Maternal Depression During Pregnancy and Recent Stress With Brain Age Among Adult Offspring. JAMA Netw. Open.

[41] Borkent J., Ioannou M., Laman J.D., Haarman B.C.M., Sommer I.E.C. (2022). Role of the gut microbiome in three major psychiatric disorders. Psychol. Med..

[42] Kumar A., Pramanik J., Goyal N., Chauhan D., Sivamaruthi B.S., Prajapati B.G., Chaiyasut C. (2023). Gut Microbiota in Anxiety and Depression: Unveiling the Relationships and Management Options. Pharmaceuticals.

[43] Jin X., Zhang Y., Celniker S.E., Xia Y., Mao J.H., Snijders A.M., Chang H. (2021). Gut microbiome partially mediates and coordinates the effects of genetics on anxiety-like behavior in Collaborative Cross mice. Sci. Rep..

[44] Yang J., Li Y., Wen Z., Liu W., Meng L., Huang H. (2021). Oscillospira—A candidate for the next-generation probiotics. Gut Microbes.

[45] Yay E., Yilmaz M., Toygar H., Balci N., Rivas C.A., Kilic B.B., Zirh A., Paster B., Kantarci A. (2023). Parkinson′s disease alters the composition of subgingival microbiome. J. Oral Microbiol..

[46] Kim S.Y., Woo S.Y., Raza S., Ho D., Jeon S.W., Chang Y., Ryu S., Kim H.L., Kim H.N. (2023). Association between gut microbiota and anxiety symptoms: A large population-based study examining sex differences. J. Affect. Disord..

[47] Kraaij R., Schuurmans I.K., Radjabzadeh D., Tiemeier H., Dinan T.G., Uitterlinden A.G., Hillegers M., Jaddoe V.W.V., Duijts L., Moll H. (2023). The gut microbiome and child mental health: A population-based study. Brain Behav. Immun..

[48] Butler M.I., Bastiaanssen T.F.S., Long-Smith C., Morkl S., Berding K., Ritz N.L., Strain C., Patangia D., Patel S., Stanton C. (2023). The gut microbiome in social anxiety disorder: Evidence of altered composition and function. Transl. Psychiatry.

[49] Ma K., Guo L., Xu A., Cui S., Wang J.H. (2016). Molecular Mechanism for Stress-Induced Depression Assessed by Sequencing miRNA and mRNA in Medial Prefrontal Cortex. PLoS ONE.

[50] Reininghaus E.Z., Platzer M., Kohlhammer-Dohr A., Hamm C., Morkl S., Bengesser S.A., Fellendorf F.T., Lahousen-Luxenberger T., Leitner-Afschar B., Schoggl H. (2020). PROVIT: Supplementary Probiotic Treatment and Vitamin B7 in Depression-A Randomized Controlled Trial. Nutrients.

[51] Brushett S., Gacesa R., Vila A.V., Gois M.F.B., Andreu-Sanchez S., Swarte J.C., Klaassen M.A.Y., Collij V., Sinha T., Bolte L.A. (2023). Gut feelings: The relations between depression, anxiety, psychotropic drugs and the gut microbiome. Gut Microbes.

[52] Thomann A.K., Wüstenberg T., Wirbel J., Knoedler L.L., Thomann P.A., Zeller G., Ebert M.P., Lis S., Reindl W. (2022). Depression and fatigue in active IBD from a microbiome perspective-a Bayesian approach to faecal metagenomics. BMC Med..

[53] Yang B., Ryu J.S., Rim C., Shin J.U., Kwon M.S. (2022). Possible role of arginase 1 positive microglia on depressive/anxiety-like behaviors in atopic dermatitis mouse model. Arch. Pharm. Res..

[54] Elgun S., Kumbasar H. (2000). Increased serum arginase activity in depressed patients. Prog. Neuropsychopharmacol. Biol. Psychiatry.

[55] Stiernborg M., Debelius J.W., Yang L.L., Skott E., Millischer V., Giacobini M., Melas P.A., Boulund F., Lavebratt C. (2023). Bacterial gut microbiome differences in adults with ADHD and in children with ADHD on psychostimulant medication. Brain Behav. Immun..

[56] Fresquet V., Williams L., Raushel F.M. (2004). Mechanism of cobyrinic acid a,c-diamide synthetase from Salmonella typhimurium LT2. Biochemistry.

[57] Maggio-Hall L.A., Escalante-Semerena J.C. (1999). In vitro synthesis of the nucleotide loop of cobalamin by Salmonella typhimurium enzymes. Proc. Natl. Acad. Sci. USA.

[58] Roper J.M., Raux E., Brindley A.A., Schubert H.L., Gharbia S.E., Shah H.N., Warren M.J. (2000). The enigma of cobalamin (vitamin B) biosynthesis in: Identification and characterization of a functional corrin pathway. J. Biol. Chem..

[59] Scott A.I., Roessner C.A., Stolowich N.J., Spencer J.B., Min C., Ozaki S.I. (1993). Biosynthesis of vitamin B12. Discovery of the enzymes for oxidative ring contraction and insertion of the fourth methyl group. FEBS Lett..

[60] Warren M.J., Raux E., Schubert H.L., Escalante-Semerena J.C. (2002). The biosynthesis of adenosylcobalamin (vitamin B12). Nat. Prod. Rep..

[61] Moore S.J., Lawrence A.D., Biedendieck R., Deery E., Frank S., Howard M.J., Rigby S.E., Warren M.J. (2013). Elucidation of the anaerobic pathway for the corrin component of cobalamin (vitamin B12). Proc. Natl. Acad. Sci. USA.

[62] Xu Y.Y., Xiang S.S., Ye K., Zheng Y.Q., Feng X., Zhu X., Chen J., Chen Y.W. (2018). Cobalamin (Vitamin B12) Induced a Shift in Microbial Composition and Metabolic Activity in an Colon Simulation. Front. Microbiol..

[63] Guetterman H.M., Huey S.L., Knight R., Fox A.M., Mehta S., Finkelstein J.L. (2022). Vitamin B-12 and the Gastrointestinal Microbiome: A Systematic Review. Adv. Nutr..

[64] Sangle P., Sandhu O., Aftab Z., Anthony A.T., Khan S. (2020). Vitamin B12 Supplementation: Preventing Onset and Improving Prognosis of Depression. Cureus J. Med. Sci..

[65] Osadchiy V., Martin C.R., Mayer E.A. (2019). The Gut-Brain Axis and the Microbiome: Mechanisms and Clinical Implications. Clin. Gastroenterol. Hepatol..

[66] Rieder R., Wisniewski P.J., Alderman B.L., Campbell S.C. (2017). Microbes and mental health: A review. Brain Behav. Immun..

[67] Dinan T.G., Cryan J.F. (2017). Brain-Gut-Microbiota Axis and Mental Health. Psychosom. Med..

[68] Foster J.A., Rinaman L., Cryan J.F. (2017). Stress & the gut-brain axis: Regulation by the microbiome. Neurobiol. Stress.

[69] Keller J., Gomez R., Williams G., Lembke A., Lazzeroni L., Murphy G.M., Schatzberg A.F. (2017). HPA axis in major depression: Cortisol, clinical symptomatology and genetic variation predict cognition. Mol. Psychiatry.

[70] Winter G., Hart R.A., Charlesworth R.P.G., Sharpley C.F. (2018). Gut microbiome and depression: What we know and what we need to know. Rev. Neurosci..

[71] Doran C.M., Kinchin I. (2019). A review of the economic impact of mental illness. Aust. Health Rev..

[72] Rhee S.J., Kim H., Lee Y., Lee H.J., Park C.H.K., Yang J., Kim Y.K., Ahn Y.M. (2021). The association between serum microbial DNA composition and symptoms of depression and anxiety in mood disorders. Sci. Rep..

[73] Cipriani A., Furukawa T.A., Salanti G., Chaimani A., Atkinson L.Z., Ogawa Y., Leucht S., Ruhe H.G., Turner E.H., Higgins J.P.T. (2018). Comparative Efficacy and Acceptability of 21 Antidepressant Drugs for the Acute Treatment of Adults With Major Depressive Disorder: A Systematic Review and Network Meta-Analysis. Focus.

[74] Rothschild D., Weissbrod O., Barkan E., Kurilshikov A., Korem T., Zeevi D., Costea P.I., Godneva A., Kalka I.N., Bar N. (2018). Environment dominates over host genetics in shaping human gut microbiota. Nature.

[75] Deschasaux M., Bouter K.E., Prodan A., Levin E., Groen A.K., Herrema H., Tremaroli V., Bakker G.J., Attaye I., Pinto-Sietsma S.J. (2018). Depicting the composition of gut microbiota in a population with varied ethnic origins but shared geography. Nat. Med..

[76] Sun Y., Zuo T., Cheung C.P., Gu W.X., Wan Y.T., Zhang F., Chen N., Zhan H., Yeoh Y.K., Niu J.K. (2021). Population-Level Configurations of Gut Mycobiome Across 6 Ethnicities in Urban and Rural China. Gastroenterology.

[77] Hadfield K., Akyirem S., Sartori L., Abdul-Latif A.M., Akaateba D., Bayrampour H., Daly A., Hadfield K., Abiiro G.A. (2022). Measurement of pregnancy-related anxiety worldwide: A systematic review. BMC Pregnancy Childbirth.

[78] Brunton R.J., Dryer R., Saliba A., Kohlhoff J. (2015). Pregnancy anxiety: A systematic review of current scales. J. Affect. Disord..

[79] Simpson C.A., Adler C., du Plessis M.R., Landau E.R., Dashper S.G., Reynolds E.C., Schwartz O.S., Simmons J.G. (2020). Oral microbiome composition, but not diversity, is associated with adolescent anxiety and depression symptoms. Physiol. Behav..

[80] Ritchie G., Strodl E., Parham S., Bambling M., Cramb S., Vitetta L. (2023). An exploratory study of the gut microbiota in major depression with anxious distress. J. Affect. Disord..

[81] Xiong R.G., Li J.H., Cheng J., Zhou D.D., Wu S.X., Huang S.Y., Saimaiti A., Yang Z.J., Gan R.Y., Li H.B. (2023). The Role of Gut Microbiota in Anxiety, Depression, and Other Mental Disorders as Well as the Protective Effects of Dietary Components. Nutrients.

