# Supplementary material for: Exploring the Association between Anxiety, Depression, and Gut Microbiota during Pregnancy: Findings from a Pregnancy Cohort Study in Shijiazhuang, Hebei Province, China
Source: Nutrients. 2024 May 12;16(10):1460. doi: 10.3390/nu16101460 (PMC11123899; doi:10.3390/nu16101460)
Supplement: Supplementary file 1 [file nutrients-16-01460-s001.zip › nutrients-2967446-supplementary.pdf]

nutrients MDPI

Supplementary materials

Table S1: The List of Species Significantly Associated with Anxiety and Depression in Unadjusted Model

**Feature**	**Variable**	**Estimate**	**Standard Error**	***P***	***Q***
Butyricimonas_sp._An62	Depression Score	0.000002	0.0000004	<0.001	0.069
Oscillibacter_sp._KLE_1745	Depression Score	0.000003	0.0000007	<0.001	0.131
Oscillibacter_sp._KLE_1728	Anxiety Score	0.000013	0.0000008	<0.001	0.167
Oscillibacter_sp._KLE_1745	Anxiety Score	0.000004	0.0000008	<0.001	0.167
Oscillibacter_sp._PEA192	Anxiety Score	0.000024	0.0000031	<0.001	0.167

Table summarizing the species significantly associated with anxiety and depression. The generalized linear mixed-effects regressions conducted using MaAsLin2 were adjusted for time, with subject as a random intercept.

Table S2: The List of Species Significantly Associated with Anxiety and Depression in Adjusted Model

**Feature**	**Variable**	**Estimate**	**Standard Error**	***P***	***Q***
Oscillibacter_sp._KLE_1745	Anxiety Score	0.0000043	0.0000009	<0.001	0.003
Oscillibacter_sp._PEA192	Anxiety Score	0.0000258	0.0000056	<0.001	0.010
Oscillibacter_sp._KLE_1728	Anxiety Score	0.0000145	0.0000032	<0.001	0.011
Oscillibacter_sp._KLE_1745	Depression Score	0.0000032	0.0000007	<0.001	0.015
Oscillospiraceae_bacterium_VE202_24	Anxiety Score	0.0000043	0.0000010	<0.001	0.018
Oscillibacter_sp._KLE_1745	Group_Anxiety	0.0000977	0.0000221	<0.001	0.018
Treponema_socranskii	Anxiety Score	0.0000004	0.0000001	<0.001	0.040
Oscillibacter_sp._KLE_1728	Group_Anxiety	0.0003350	0.0000808	<0.001	0.046
Neocallimastix_californiae	Group_Anxiety	0.0000139	0.0000036	<0.001	0.065
Alistipes_sp._AL_1	Group_Anxiety	0.0001564	0.0000393	<0.001	0.065
Oscillibacter_sp._KLE_1728	Depression Score	0.0000107	0.0000027	<0.001	0.069
Bacteroides_sp._41_26	Depression Score	0.0000009	0.0000002	<0.001	0.069
Firmicutes_bacterium_CAG_137	Group_Anxiety	0.0003328	0.0000839	<0.001	0.071
Oscillibacter_sp._PEA192	Depression Score	0.0000188	0.0000047	<0.001	0.075
Bacteroides_nordii	Depression Score	0.0000217	0.0000055	<0.001	0.077
Oscillospiraceae_bacterium_VE202_24	Depression Score	0.0000032	0.0000008	<0.001	0.078
Butyricimonas_sp._An62	Depression Score	0.0000016	0.0000004	<0.001	0.086
Dysgonomonas_mossii	Group_Anxiety	0.0000063	0.0000013	<0.001	0.086
Firmicutes_bacterium_CAG_137_57_8	Group_Anxiety	0.0000802	0.0000207	<0.001	0.087
Ruminococcus_sp._AM41_10BH	Anxiety Score	0.0000009	0.0000002	<0.001	0.091
Bacteroides_sp._AR20	Group_Depression	0.0000644	0.0000167	<0.001	0.101
Bacteroides_sp._HPS0048	Depression Score	0.0000090	0.0000024	<0.001	0.108
Oscillibacter_sp._KLE_1745	Group_Depression	0.0000758	0.0000201	<0.001	0.125
Prevotella_sp._P5_108	Group_Depression	0.0000142	0.0000039	<0.001	0.129
Faecalibacterium_virus_Brigit	Depression Score	0.0000006	0.0000002	<0.001	0.136
Desulfotomaculum_aeronauticum	Group_Anxiety	0.0000090	0.0000025	<0.001	0.141
Bacillus_saganii	Group_Anxiety	0.0000052	0.0000012	<0.001	0.153
Acetivibrio_thermocellus	Group_Anxiety	0.0000151	0.0000042	<0.001	0.173

Bacillus_saganii	Anxiety Score	0.0000002	0.0000000	<0.001	0.177
Oscillibacter_sp._PEA192	Group_Anxiety	0.0005269	0.0001461	<0.001	0.180
Clostridium_sp._HGF2	Group_Anxiety	0.0000450	0.0000127	<0.001	0.200
Ruminococcus_sp._OF03_6AA	Anxiety Score	0.0000010	0.0000003	0.001	0.209
Oscillospiraceae_bacterium_VE202_24	Group_Anxiety	0.0000880	0.0000251	0.001	0.219
Ruminococcus_sp._AM41_10BH	Group_Anxiety	0.0000204	0.0000059	0.001	0.222
Butyricimonas_synergistica	Depression Score	0.0000023	0.0000007	0.001	0.246

Table S2 summarizing the species significantly associated with anxiety and depression. The generalized linear mixed-effects regressions conducted using MaAsLin2 were adjusted for time, age, parity, pre-pregnancy BMI, history of smoking, alcohol drinking before pregnancy, energy intake, physical activity level, probiotics, and prebiotics intake, with subject as a random intercept. Group_Anxiety represents grouping based on anxiety status (Anxiety/Non-Anxiety), while Group_Depression represents grouping based on depression status(Depression/Non-Depression).

Table S3: Functional Pathways and Their Correlation with Anxiety/Depression Scores and Anxiety/Non-anxiety and Depression/Non-depression Groupings in Unadjusted Model

**Pathway**	**Variable**	**Estimate**	**Standard Error**	***P***	***Q***
ko00430: Taurine.and.hypotaurine.metabolism	Anxiety Score	0.0000035	0.0000013	0.007	0.638
ko00565: Ether.lipid.metabolism	Anxiety Score	0.0000006	0.0000003	0.022	0.723
ko00760: Nicotinate.and.nicotinamide.metabolism	Anxiety Score	0.0000061	0.0000030	0.045	0.723
ko00130: Ubiquinone.and.other.terpenoid.quinone.biosynthesis	Anxiety Score	0.0000098	0.0000049	0.047	0.723
ko00592: alpha.Linolenic.acid.metabolism	Anxiety Score	0.0000011	0.0000006	0.057	0.723
ko00760: Nicotinate.and.nicotinamide.metabolism	Depression Score	0.0000062	0.0000025	0.014	0.607
ko00430: Taurine.and.hypotaurine.metabolism	Depression Score	0.0000026	0.0000011	0.014	0.607
ko00620: Pyruvate.metabolism	Depression Score	0.0000100	0.0000043	0.021	0.619
ko00565: Ether.lipid.metabolism	Depression Score	0.0000005	0.0000002	0.023	0.619
ko00860: Porphyrin.and.chlorophyll.metabolism	Depression Score	-0.0000145	0.0000066	0.029	0.619
ko00730: Thiamine.metabolism	Group_Anxiety	-0.0002334	0.0000877	0.008	0.519
ko00627: Aminobenzoate.degradation	Group_Anxiety	0.0000876	0.0000367	0.018	0.519
ko00620: Pyruvate.metabolism	Group_Anxiety	0.0003038	0.0001290	0.019	0.519
ko01054: Nonribosomal.peptide.structures	Group_Anxiety	0.0000245	0.0000105	0.021	0.519
ko00430: Taurine.and.hypotaurine.metabolism	Group_Anxiety	0.0000756	0.0000327	0.022	0.519
ko00860: Porphyrin.and.chlorophyll.metabolism	Group_Depression	-0.0006189	0.0001803	0.001	0.327
ko00760: Nicotinate.and.nicotinamide.metabolism	Group_Depression	0.0002015	0.0000690	0.004	0.403
ko00591: Linoleic.acid.metabolism	Group_Depression	0.0000073	0.0000025	0.004	0.403
ko00430: Taurine.and.hypotaurine.metabolism	Group_Depression	0.0000855	0.0000295	0.004	0.403
ko00440: Phosphonate.and.phosphinate.metabolism	Group_Depression	0.0001304	0.0000539	0.017	0.487

For each variable, the top five pathways are selected for reporting, as determined by ascending *Q*-values and *P*-values.

Table S4: Functional Pathways and Their Correlation with Anxiety/Depression Scores and Anxiety/Non-anxiety and Depression/Non-depression Groupings in Adjusted Model

**Pathway**	**Variable**	**Estimate**	**Standard Error**	***P***	***Q***
ko00430:Taurine.and.hypotaurine.metabolism	Anxiety Score	0.0000033	0.0000013	0.014	0.977
ko00565:Ether.lipid.metabolism	Anxiety Score	0.0000006	0.0000003	0.029	0.977
ko00591:Linoleic.acid.metabolism	Anxiety Score	0.0000002	0.0000001	0.035	0.977
ko03450:Non.homologous.end.joining	Anxiety Score	0.0000003	0.0000001	0.035	0.977
ko00130: Ubiquinone.and.other.terpenoid.quinone.biosynthesis	Anxiety Score	0.0000108	0.0000052	0.039	0.977
ko00760:Nicotinate.and.nicotinamide.metabolism	Depression Score	0.0000068	0.0000026	0.009	0.968
ko00430:Taurine.and.hypotaurine.metabolism	Depression Score	0.0000025	0.0000011	0.026	0.968
ko00565:Ether.lipid.metabolism	Depression Score	0.0000005	0.0000002	0.029	0.968
ko03020:RNA.polymerase	Depression Score	-0.0000036	0.0000017	0.034	0.968
ko00020:Citrate.cycle:TCA.cycle.	Depression Score	0.0000105	0.0000050	0.038	0.968
ko00730:Thiamine.metabolism	Group_Anxiety	-0.0002453	0.0000902	0.007	0.967
ko01054:Nonribosomal.peptide.structures	Group_Anxiety	0.0000287	0.0000108	0.009	0.967
ko00903:Limonene.and.pinene.degradation	Group_Anxiety	0.0000448	0.0000173	0.011	0.967
ko00627:Aminobenzoate.degradation	Group_Anxiety	0.0000965	0.0000382	0.013	0.967
ko00590:Arachidonic.acid.metabolism	Group_Anxiety	0.0000495	0.0000198	0.013	0.967
ko00591:Linoleic.acid.metabolism	Group_Depression	0.0000081	0.0000026	0.002	0.979
ko00860:Porphyrin.and.chlorophyll.metabolism	Group_Depression	-0.0005583	0.0001864	0.003	0.979
ko00760:Nicotinate.and.nicotinamide.metabolism	Group_Depression	0.0001948	0.0000709	0.007	0.979
ko00430:Taurine.and.hypotaurine.metabolism	Group_Depression	0.0000760	0.0000306	0.014	0.979
ko00909:Sesquiterpenoid.and.triterpenoid.biosynthesis	Group_Depression	0.0000063	0.0000026	0.017	0.979

For each variable, the top five pathways are selected for reporting, as determined by ascending *Q*-values and *P*-values.

Table S5: The List of Enzymes Significantly Associated with Anxiety and Depression in Unadjusted Model

**Feature**	**Variable**	**Estimate**	**Standard Error**	**P**	**Q**
EC.2.1.1.195: cobalt-precorrin-5B (C1)-methyltransferase	Group_Depression	-0.0001280	0.0000323	<0.001	0.109
EC.2.7.8.26: adenosylcobinamide-GDP ribazoletransferase	Group_Depression	-0.0001146	0.0000285	<0.001	0.109
EC.6.3.1.10: adenosylcobinamide-phosphate synthase	Group_Depression	-0.0001255	0.0000313	<0.001	0.109
EC.6.3.5.9.6.3.5.11: cobyrinic acid a,c-diamide synthase	Group_Depression	-0.0001361	0.0000327	<0.001	0.109
EC.2.1.1.131: precorrin-3B C17-methyltransferase	Group_Depression	-0.0001353	0.0000357	<0.001	0.152
EC.6.3.5.10: adenosylcobyric acid synthase (glutamine-hydrolyzing)	Group_Depression	-0.0001131	0.0000303	<0.001	0.165

EC.3.7.1.12: cobalt-precorrin 5A hydrolase	Group_Depression	-0.0001341	0.0000366	<0.001	0.186
EC.3.5.3.1: arginase	Group_Anxiety	0.0000772	0.0000191	<0.001	0.194

Figure S1:

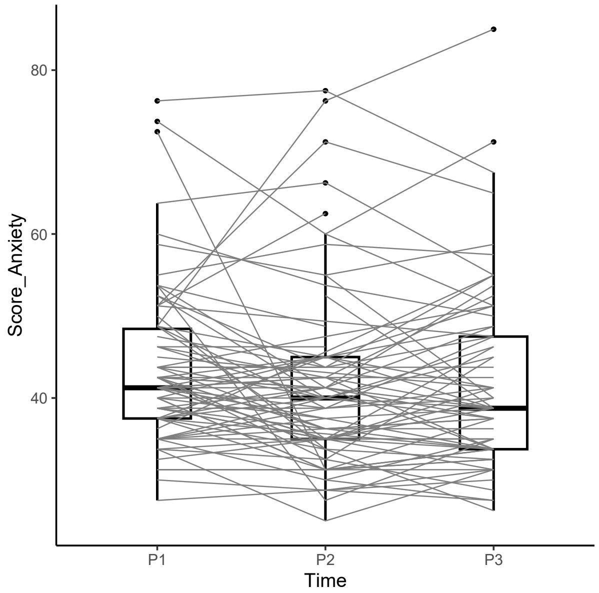

Figure S1: Temporal Trends in Anxiety Scores Across Multiple Time Points. The figure illustrates the changes in anxiety scores of research subjects at different time points. Each data point represents an individual research subject's data at a specific time point, and the data for the same research subject across different time points is connected by a line. The central bold line within each box represents the median, indicating the middle value where half the data points are above and half are below. The edges of the box delineate the upper and lower quartiles, marking the 75th and 25th percentiles, respectively.

Figure S2:

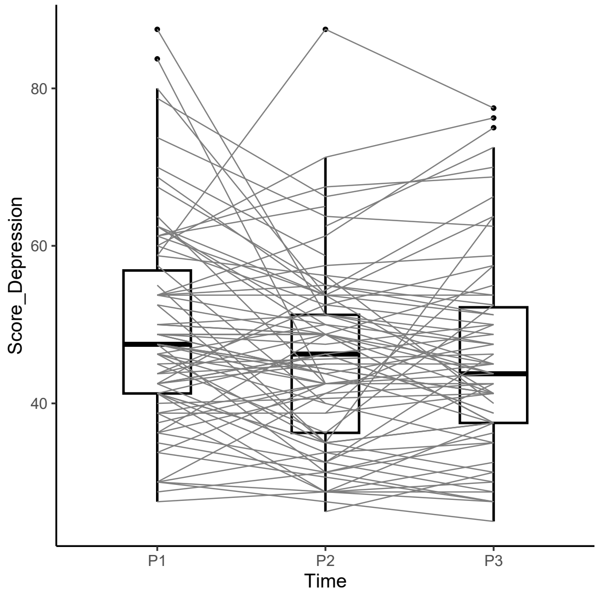

Figure S2. Temporal Trends in Depression Scores Across Multiple Time Points. The figure illustrates the changes in depression scores of research subjects at different time points. Each data point represents an individual research subject's data at a specific time point, and the data for the same research subject across different time points is connected by a line. The central bold line within each box represents the median, indicating the middle value where half the data points are above and half are below. The edges of the box delineate the upper and lower quartiles, marking the 75th and 25th percentiles, respectively.
